# Evaluation of the Effects of* Aedes* Vector Indices and Climatic Factors on Dengue Incidence in Gampaha District, Sri Lanka

**DOI:** 10.1155/2019/2950216

**Published:** 2019-01-31

**Authors:** N. D. A. D. Wijegunawardana, Y. I. N. Silva Gunawardene, T. G. A. N. Chandrasena, R. S. Dassanayake, N. W. B. A. L. Udayanga, W. Abeyewickreme

**Affiliations:** ^1^Molecular Medicine Unit, Faculty of Medicine, University of Kelaniya, Ragama, Sri Lanka; ^2^Department of Parasitology, Faculty of Medicine, University of Kelaniya, Ragama, Sri Lanka; ^3^Department of Chemistry, Faculty of Science, University of Colombo, Sri Lanka; ^4^Department of Bio-Systems Engineering, Faculty of Agriculture & Plantation Management, Wayamba University of Sri Lanka, Sri Lanka; ^5^Department of Parasitology, Faculty of Medicine, General Sir John Kotelawala Defence University, Sri Lanka

## Abstract

Constant monitoring of* Aedes* vector indices such as* Aedes* mosquito abundance and ovitrap data is important for the control of dengue epidemics. Therefore, the current study attempted to evaluate the effect of larval and climatic factors on the incidence of dengue outbreaks in the Gampaha district. Based on the distribution of previously reported dengue cases, 34 households in Narangodapaluwa PHI area, Ragama, Sri Lanka, were selected randomly, and entomological surveillance was done fortnightly using adult mosquito catches and larval surveillance techniques for a period of two years. Further, weekly ovitrap surveillance was conducted for one year, by maintaining four ovitraps in a single house, two indoors and two outdoors at ground and at a height of 1.5–2 m. Based on the findings, larval indices, namely, Breteau index (BI), House index (HI), and Container index (CI), were calculated, along with the Ovitrap index (OI). The study area was positive for* Ae. albopictus* with an adult capturing range of 1~15/34 households. BI initially remained < 3%, which subsequently decreased up to 0. No significant difference in OI was found between the ovitraps placed at ground level and at a height of 1.5-2m (p>0.05), 95% level of confidence. The OI varied from 56.9% to 94.7% during the study period of 12 months, indicating two peaks at the monsoons. Statistics of one-way ANOVA revealed a significant difference in the monthly OI during the study period (p≤0.001) with two peaks representing the monsoonal rainfall patterns. Pearson's correlation analysis revealed that the association between dengue cases and larval indices (BI, CI, HI, and OI) and meteorological parameters was not significant (p<0.05). Migration of mosquitoes and patients could be considered as possible factors affecting the absence of a significant relationship.

## 1. Introduction

Dengue fever is the most important arboviral disease as 40% of the population of the world in 128 countries has been predicted to be at risk of infection with an estimated 50–100 million cases of dengue occurring annually [1, 2]. Despite the control measures implemented, there has been no significant reduction in the number of dengue cases in Sri Lanka, with an average number of cases ranging from 30,000 to 35,000 annually [3]. However, the mortality rate due to dengue has been reduced from around 1% in 2009 to 0.28% in 2013. Gampaha remained the second most high-risk area for dengue within Sri Lanka.

Dengue viruses (DENV) are primarily transmitted by the infectious bites of female* Ae. aegypti* mosquitoes and to a much lesser extent by* Ae. albopictus* [4]. However, few studies in several other countries have shown that* Ae. albopictus* could also play an important role at local level [5].* Aedes *mosquitoes live in close proximity to people and play a major role in transmission of dengue (DENV) viruses.

At present with no effective vaccines against dengue fever [6], vector control plays a major role in curbing the incidence of the disease worldwide [7]. The vector control approach emphasizes the need for vector surveillance, with the objectives of maintaining* Ae. aegypti* and* Ae. albopictus* populations below or close to the transmission threshold values, slowing dengue virus transmission, and reducing sequential infections with heterologous serotypes that can increase the incidence of serious disease.

*Ae. aegypti* and* Ae. albopictus* use natural and artificial water-holding containers (e.g., tree holes, used tires, plastic containers, clogged gutters) to lay their eggs. After hatching, larvae grow and develop into pupae and subsequently into terrestrial, flying adult mosquitoes. The prevention or reduction of transmission of DENV is dependent on the control of mosquito vectors and limiting of human-mosquito contact. Mosquito surveillance has become a key component of any local integrated vector management program with the goal of quantifying human risk by determining local vector abundance.

Mosquito-based surveillance mainly focuses on the collection of specimens, including eggs, larvae, pupae, or adults. Ovitrap survey is the most widely practiced method of egg collection. Ovitraps are small metal, glass, or plastic containers, usually dark in colour, containing water and a substrate (wood, seed germination paper, cloth, plant gel) allowing the female mosquitoes to lay their eggs [8]. Ovitraps take advantage of the fact that gravid* Ae. aegypti* and* Ae. albopictus* females lay their eggs in artificial containers. Adequate sampling requires regular (weekly) trapping at fixed sites, representative of their habitat types, in the community. Ovitraps should not be deployed in the field for more than a week at a time, because they could become breeding sites for production of adult mosquitoes [9].

Conducting of ovitrap surveys has several advantages, inexpensiveness, easy deployability, and noninvasiveness. A small number of ovitraps is usually adequate to determine the presence of vectors, whereby less than 100 ovitraps can reliably estimate the vector abundance in a large urban neighbourhood [10]. Typically, one ovitrap is placed per city block. Lastly, ovitrap data is easy to analyse and is usually expressed as the percentage of positive ovitraps (ovitraps with eggs). The mean number of eggs per ovitrap can be used to estimate the abundance of adult mosquitoes [9]. However, according to recent evidence, the average number of eggs per ovitrap provides only an indirect estimation of the abundance of adult mosquitoes. The association between ovitrap data and population size needs to be identified very carefully, as often it remains less straightforward. Since a single female might lay up to 100 eggs on a single gonotrophic cycle and due to the skip-ovipositing nature of* Aedes* females, the estimates of adult population based on the number of eggs on ovitraps have a wider uncertainty according to the foresaid explanation. For example, an ovitrap with 500 eggs might be visited by 5-500 females. This large uncertainty makes estimation of adult mosquito population by ovitraps, very fragile and limited.

For accurate mosquito-based surveys, surveillance for immature stages (larvae and pupae) and adult mosquitoes should also be performed along with ovitrap surveys, by using different approaches [9]. Therefore, the objective of this study was to identify factors leading to the high occurrence of dengue fever (DF) within the study area that reported the highest number of DF cases within the district of Gampaha, while identifying the most preferred types of container habitats by* Aedes* vectors to facilitate vector control efforts. Furthermore, data have been used to develop detailed maps to track mosquito breeding sites, in which* Ae. aegypti* or* Ae. albopictus* were detected within the area. This could be useful to determine mosquito population and identify geographic areas of high mosquito abundance (hot-spots), where control measures are essential. The outcome of this study would be of use in identifying primary/secondary mosquito vectors, factors responsible for the prevailing high incidence of dengue in Narangodapaluwa PHI area, which would facilitate precise targeting of control measures.

## 2. Methodology

### 2.1. Study Site

**Figure 1:**
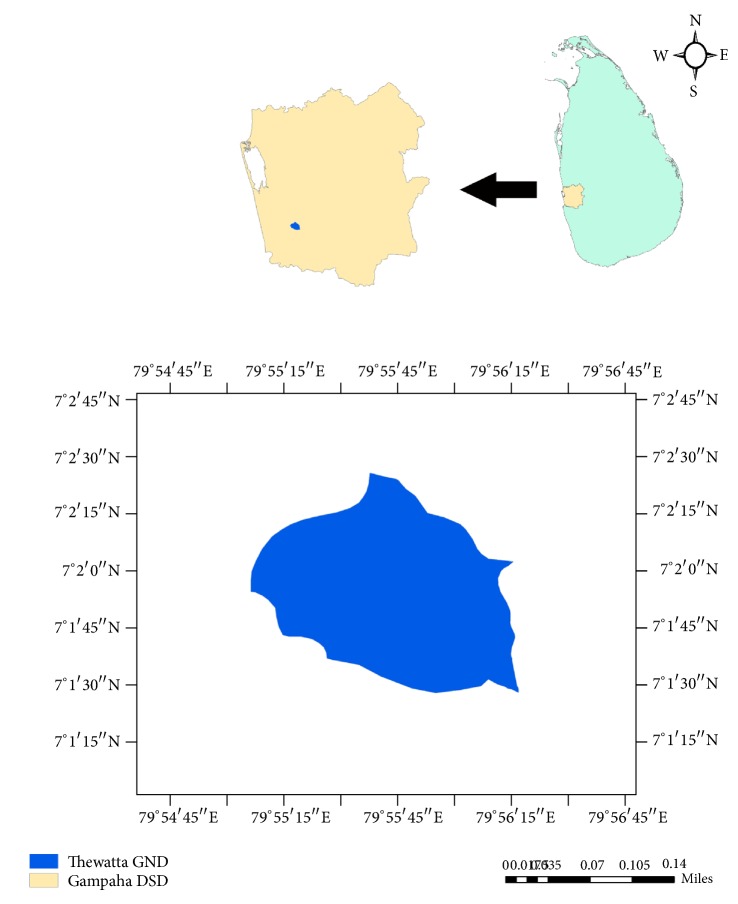
Location of the Narangodapaluwa PHI area within the Gampaha district.

The district of Gampaha has remained the second high-risk district in Sri Lanka for dengue throughout the last five years. In 2018, Gampaha district accounted for 11.35% (n=5,857) of 51,591 suspected dengue cases that have been reported from Sri Lanka [11]. From the entire Gampaha district, Ragama Medical Officer of Health area, which remains a prominent risk area for dengue, was selected as the study locality. Since surveying a large area with an unequal dengue case distribution was not feasible, Narangodapaluwa PHI area (Figure 1) located within the Ragama MOH area (7°2′51′′N and longitude of 79°56′0′′E) was selected based on the convenience of sampling [11, 12].

The Narangodapaluwa PHI area hosts a total population of 4, 121 and has reported 268 suspected dengue cases within 2014. Therefore, households located within the Narangodapaluwa PHI area that had reported dengue cases within 2014, were selected as the sampling sites. Selection of households for the ovitrap survey was purposely carried out based on the judgmental sampling method and out of 268 total number of DF positive cases reported to the Ragama MOH in year 2014 [12]. The selection criterion also included location of the houses in the close proximity of the study area on the above MOH area (Figure 1), due to practical feasibility of regular sample collection and handling. Accordingly, 42 DF positive patients in 34 households were selected for the study. The study areas included households with moderate facilities, with 2-3 rooms in common. The geographic locations of the households were recorded by using GPS receivers and were marked on a map by using ArcGIS (version 10.2).

### 2.2. Study Duration

A two-year mosquito adult and larvae survey was conducted from September 2014 to September 2016 fortnightly in the Ragama MOH area. Meanwhile, an ovitrap survey was conducted at weekly intervals within the same study area from June 2015 to May 2016.

### 2.3. Mosquito-Based Surveillance: Specimen Collection

#### 2.3.1. Adult Mosquito Surveys

Adult mosquito surveys were conducted once in two weeks in the above selected 34 households in the Narangodapaluwa PHI area, by using both mechanically modified CDC backpack aspirators (Model 1412, John W. Hock Company, USA) and standard manual mouth aspirators (Model 612, John W. Hock Company, USA), which includes a 0.3-micron HEPA filter with screen to stop insect particles from entering to the mouth. Both these aspirators were used simultaneously and the same premises were inspected during the two-year sampling by a trained team of field entomological assistants.

#### 2.3.2. Immature Stage (Larvae and Pupae) Surveys

During the surveys, all types of water-holding containers were inspected for the presence of* Aedes* larvae/pupae, and the number and type of container(s) positive for* Aedes* larvae or pupae were recorded. Presence of* Aedes* larvae in natural breeding habitats was also recorded. While larval indices were used to quantify vector breeding potential in each cluster, the pupal counts were taken as a proxy for adult vector abundance. To calculate Breteau index, at least 100 houses/premises were surveyed. In addition, potential and most preferred breeding sites for each mosquito species were identified and recorded.

#### 2.3.3. Ovitrap Surveys

*(a) Initial 6-Month Ovitrap Survey. *A pilot ovitrap survey of six months was conducted to assess the suitable position for placing the ovitraps for dengue vector mosquito surveillance. As mentioned earlier, thirty-four households in the Ragama Medical Officer of Health area in Gampaha district were selected to conduct the pilot ovitrap survey during the period of June to November 2015 to select the most appropriate positioning height and location of the ovitrap. The conventional black plastic coded ovitraps (3.2 x 2.7 cm) with plywood paddles (4 x 0.5 cm) placed over the upper rim were used to collect the immature stages of* Aedes* mosquitoes. A total of 136 ovitraps (four per house, two of which were placed indoors and the other two outdoors) were used. Of the two ovitraps that were placed indoors and outdoors, one was hung at a height of 1.5–2m, while the other was kept on the ground. In positioning ovitraps, the outdoor ones were kept 3m away from the house, and the indoor ovitraps were placed in the living room in close proximity to racks/hanging clothes, partially shaded places, or kitchen area (especially under the water sink) as such areas are more attractive to the mosquitoes for resting due to shade and humid condition. Following collection of samples each week, ovitraps were washed thoroughly and refilled with new water and a new paddle. The number of* Aedes* eggs and immature stages found in ovitraps was recorded.

*(b) Final One-Year Ovitrap Survey. *Based on the data obtained from the initial 6-month pilot ovitrap survey, ovitrap surveillance was continued using hanging ovitraps. Out of a total of 68 ovitraps, two hanging ovitraps (1 indoor and 1 outdoor) were placed at each house in the study site. Initially mentioned considerations were followed in placing the ovitraps, and sample collection and handling were done as described previously. A GIS map of the ovitrap positioning sites was developed by using ArcGIS (version 10.2). One-year ovitrap survey was conducted from June 2015 to May 2016.

### 2.4. Transporting Mosquito Samples, Identification, and Rearing

Collected eggs and larval and pupal samples were transferred to the insectary at the Molecular Medicine Unit (MMU), Faculty of Medicine, University of Kelaniya, Ragama, Sri Lanka.* Aedes* mosquitoes were sorted from the field collections and identified up to the species level by using standard keys [13]. The immature stages were reared up to adulthood followed by the taxonomic identification.

### 2.5. Collection of Meteorological Data

Monthly cumulative rainfall and minimum and maximum temperature for the period of September 2014 to September 2016, corresponding to the Henarathgoda agrometrological station, were collected from the Department of Meteorology, Colombo. The distance between meteorological station and the field site was approximately 21.2 km.

### 2.6. Data Recording and Statistical Analysis

The identified immature stages and adult mosquitoes were enumerated separately, the data were entered into Excel worksheets, and data validation was carried out based on the MOD function. However, after transferring the data into Statistical Package for Social Science (SPSS) software, data validation was carried out again before analysing the data. Entomological parameters including the dengue vector indices, namely, House index (HI), Container index (CI), and Breteau index (BI), were calculated to determine the mosquito densities along with the Ovitrap index (OI) as recommended by the WHO [14]. The relationship between the confirmed dengue cases and the meteorological parameters (rainfall, minimum and maximum temperature), collected from the Department of Meteorology, Colombo, Sri Lanka, was investigated by using the Pearson correlation analysis in SPSS (version 23).

### 2.7. Ethical Considerations

Ethical clearance for the current study was obtained from the Ethical Review Committee, Faculty of Medicine, University of Kelaniya, Ragama, Sri Lanka. Further, informed written consent was obtained from the household heads of all households agreeing to participate in entomological and ovitrap surveys.

## 3. Results

### 3.1. Adult and Larval Survey

**Figure 2:**
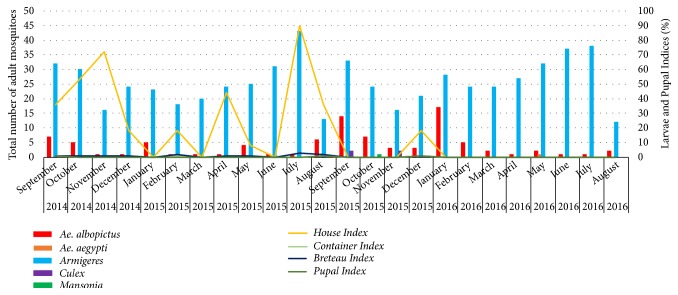
Relative abundance of adult mosquito species within study site and results of the mosquito indices (HI, CI, BI, PI) from September 2014 to August 2016.

When the relative abundance of adult mosquito species within the study site is considered, the abundance of* Armigeres subalbatus* mosquito species remained the highest, followed by* Ae. albopictus* species. Further, it was evident that Ragama PHI area in Gampaha district was positive for* Ae. albopictus* mosquito species, even at a low percentage, whereby the number of* Ae. albopictus* mosquitoes caught from 100 households at the study site varied from 1 to 17. Furthermore,* Ae. aegypti*,* Culex quinquefasciatus*, and* Mansonia uniformis* mosquito species were also found to be present in the study area in very low densities. A comparison of species specific mosquito abundance during the surveillance period at the study site is given in Figure 2. It was interesting to note that the frequency of* Aedes *adult mosquitoes increased within the study site at later stages of the surveillance, irrespective of complete removal of potential breeding sites for* Aedes* mosquitoes.

### 3.2. Immature Stage (Larvae and Pupae) Surveys

Potential breeding sites for mosquitoes were found in the study sites initially. Breteau index (BI) (number of positive containers per 100 houses inspected) calculated for each field visit always remained below 3%, while the highest value for BI was observed in July 2015 (Table 1). At later surveillance stages, complete removal of possible breeding sites was observed with BI of 0.0. During the survey, natural breeding places were identified, such as tree holes and large tree leaves (e.g.,* Tectona grandis* and* Terminalia catappa*), and some artificial containers (e.g., discarded coconut shells, discarded toy parts, and containers used for controlling the water leak and spill within the houses) play a major role in* Aedes *breeding within the study site. However, presence of breeding places was not having any notable association with the mosquito abundance, as the final stage of the survey resulted in the same mosquito density fluctuation even with the zero prevalence of breeding places.

### 3.3. Ovitrap Survey

*(i) Initial 6-Month Ovitrap Survey. *Monthly temporal variation of the average numbers of* Aedes* mosquito larvae and eggs that were found in the ovitraps is indicated in Table 1, along with the hatching rate of eggs within 48 hours. Interestingly, the average number of mosquito eggs and immature stages in ovitraps kept outside (OS) were relatively higher than those placed inside. In addition, the mosquito immature stage counts (larval, eggs present and hatched) were relatively higher in hung ovitraps than the ones kept on the ground (Table 1).

Results of the paired t test advocated the presence of a significant difference among the indoor and outdoor kept ovitraps in terms of the three variables, average number of total* Aedes *mosquito immature stages, the number of eggs present, and the number of eggs hatched (p<0.05 at 95% level of confidence). Further, the test statistics of two-way ANOVA based on the initial 6-month survey revealed that the average numbers of total* Aedes *mosquito immature stages and eggs present in hung and ground kept ovitraps were not significantly different (p>0.05), in spite of the significant difference between the outside and inside placements (p<0.05 at 95% level of confidence).

Analysis of the overall study data for missing values indicated 93.75% completeness with regard to two variables, number of ovitraps placed and collected. The missing percentage of data (6.25%) of the variable (number of ovitraps collected) was from ground placement ovitraps. Therefore, ovitraps placed at a height of 1.5-2m level (H) were selected for continuing the ovitrap surveillance in order to minimise the loss of the data caused by mechanical damage to ovitraps, thus facilitating continuous data collection.

**Figure 3:**
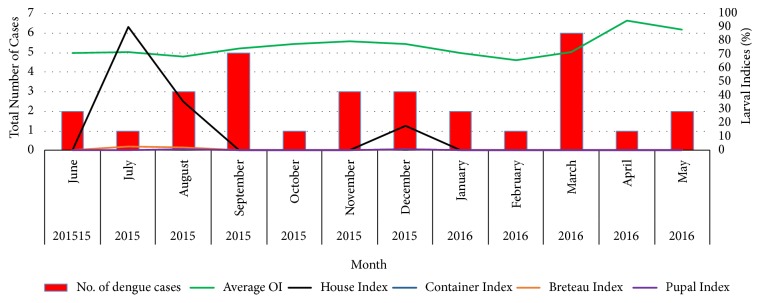
Temporal variation of larval indices (HI, CI, BI, PI) with the reported number of dengue cases from June 2015 to May 2016.

*(ii) Final One-Year Ovitrap Survey. *Mixed infestation of both* Ae. aegypti* and* Ae. albopictus* in the same ovitrap, placed indoors and outdoors, was found within the study site. The percentage of mixed infestation was very low (0.045%); out of the mixed infected ovitraps, only 10% (n=4) of eggs were from* Ae. aegypti*, and out of mixed infected 3 ovitraps, 2 were from the outside ovitraps. Therefore, average monthly OI for* Aedes *mosquitoes was calculated using data obtained for both* Aedes* species and only from the hanging ovitraps kept inside and outside during the period of June 2015 to May 2016. The distribution of numerical values gained as the average OI for* Aedes *mosquito ranged from 56.9% to 94.7% during the 12-month period of study, with a normal distribution as suggested by a Shapiro-Wilk test value of 0.901 (mean=75.28% and standard deviation=8.611). The relative abundance of* Aedes* mosquitoes depicted by the OI denoted two major peaks within the study period as September to December in 2015 and March to April in 2016 (Table 2 and Figure 3), where the average OI represented two peaks corresponding to above months. Meanwhile, the mean OI for two peaks and average OI for the rest of the months in the study year vary: 78.40%, 76.51%, and 73.20%, respectively, for the study site. However, the peak observed during March to April in 2016 was relatively higher than the other. And surprisingly, the number of* Ae. aegypti* species was slightly high within these two peak times, while the difference between two* Aedes* species was not statistically significant (p=0.451). Yet, the statistical analysis of monthly OI revealed a significant fluctuation of the average OI values at monthly intervals in accordance with ANOVA (p<0.05 at 95% level of confidence).

### 3.4. Impact of Meteorological Parameters on the Incidence of Dengue Cases and Ovitrap Index

**Figure 4:**
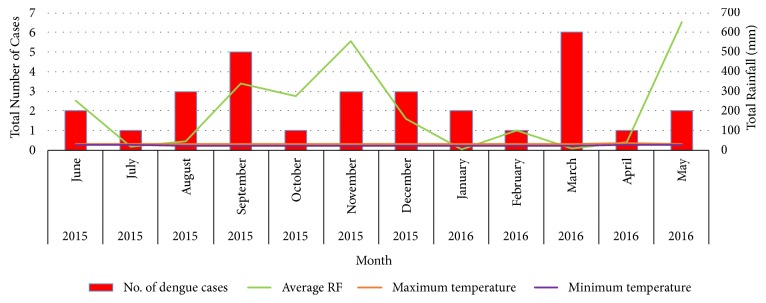
Temporal variation of meteorological parameters with the reported number of dengue cases from June 2015 to May 2016.

**Figure 5:**
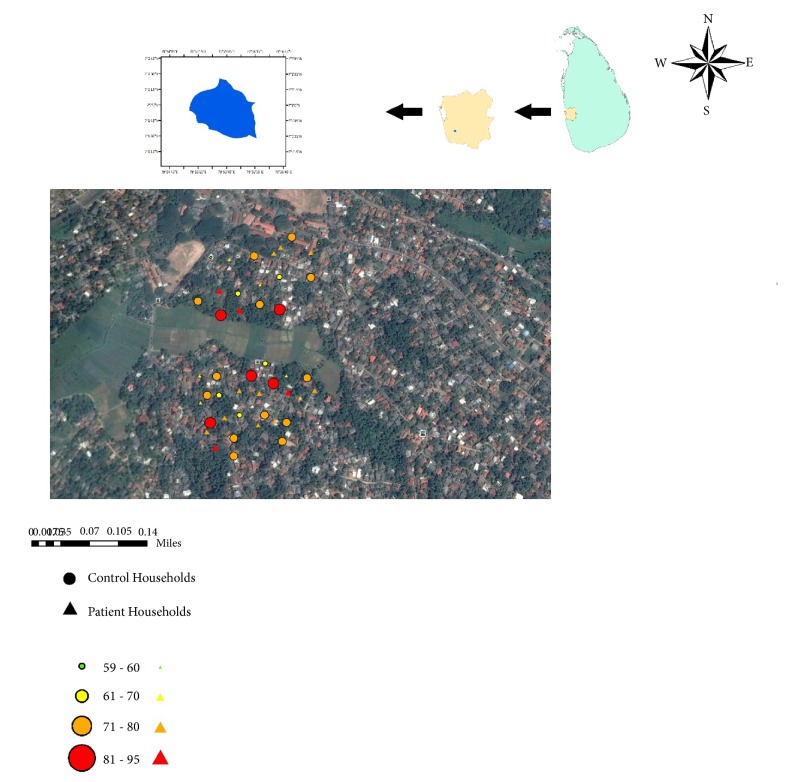
Vector distribution map constructed based on OI followed by the number of positive cases reported in the study area.

Temporal variation of the average rainfall, average maximum and minimum temperature, and number of reported dengue cases within the Narangodapaluwa MOH area is indicated in Figure 3, along with the variation of the monthly mean OI. As suggested by the results of the Pearson correlation analysis, all the meteorological parameters did not indicate any significant correlation with the number of dengue cases reported within the period of June 2015 to September 2016 (p>0.05 at 95% level of significance) at lag periods of zero, one, two, and three (Figures 3 and 4). However, cumulative rainfall denoted a positive relationship with the dengue cases, while both minimum and maximum temperature denoted a negative association (Figure 4). In case of the OI, a notable positive relationship was observed between the OI and the reported number of dengue cases at one- (Pearson Correlation Coefficient= 0.56) and two-month (Pearson Correlation Coefficient= 0.39) lag periods (Figures 3 and 5). However, similar to the earlier relationships, the association was not significant (p>0.05).

## 4. Discussion

The prevalence of dengue has been confirmed in 128 countries worldwide [15] including Sri Lanka. During the years 2015 and 2016 the number of confirmed dengue cases reported was 29, 777 and 55,150, respectively, in Sri Lanka [16]. The district of Gampaha reported the second highest number of dengue cases within the country, while Ragama MOH area also reported a considerable number of confirmed dengue cases (178) in the year 2016.

As dengue is mainly transmitted by* Aedes* mosquito species, the abundance of vector species and dengue case incidence indicate seasonal variations. Interpretation of the ovitrap data may require caution, because ovitraps compete with naturally occurring larval habitats and the estimates from oviposition surveys may not accurately reflect the abundance of gravid females under some conditions [9]. Furthermore, a considerable degree of training in microscopy is required for the accurate egg counting. In addition, the collected eggs need to be hatched, the progeny need to be reared in the laboratory, and the larvae or adults need to be identified to the species level to ensure the accuracy, which again requires trained personnel [9]. Therefore, efforts were made to study all possible entomological aspects that could affect the transmission of dengue by* Aedes* vector mosquitoes leading to conduction of adult, larval, pupae, and ovitrap surveys within the study area.

In brief this study included two-year mosquito adult and larval survey together with one-year ovitrap survey to determine the population dynamics of mosquitoes with specific interest in* Aedes* mosquitoes. Therefore, study was conducted with the aim of seeing if there is any correlation between cases and the mosquito population. From the initial ovitrap survey the optimal ovitrap positioning was identified, which reduced the loss of ovitrap data caused by mechanical damage. Thereafter, the* Aedes* vector density was assessed utilizing multiple* Aedes *mosquito sampling methods, rather than using only ovitraps. In this study an attempt was made to investigate the correlation between vector density and dengue case incidence within the Narangodapaluwa PHI area in Ragama MOH area. Accordingly, this study utilized vector indices and dengue case incidence reported in the Ragama MOH records during the study period from January 2015 to September 2016.

As highlighted by some researchers, distribution of dengue cases is highly clustered in both temporal and spatial scales. Selection of a large area might negatively influence the true nature of the relationship of the vector density and dengue case incidence as the flight range of mosquito vectors was below 800 m [17]. Therefore, this study was conducted within a PHI area with high dengue case incidence and the study duration was limited to one year. Size of the study cluster was 800 m (based on flight range of vector) and distribution of house and human density were uniform throughout the cluster.

This study utilized the dengue cases recorded by the MOH, which could slightly differ from the actual number of cases that occurred in the study area during this period. Although a positive correlation between average monthly OI and the dengue case incidence in the study area was observed, it was not significant. Further, the BI remained below 5% throughout the study period as indicated in Table 1. Therefore, the prediction of an epidemic of dengue remains difficult with this data. And to use OI as the indicator to forecast and prevent the outbreaks is insufficient as well. Therefore, OI is recommended to be used in this study to monitor the population of* Aedes* mosquitoes by detecting the presence of eggs/larvae/pupae.

The results of this survey indicated the importance of having a better knowledge of vector ecology as there was a positive association between cases and OI irrespective of the statistical significance. As displayed in Figure 3, at the end of October the onset of rainfall pattern affected the ovitrap data, reducing the OI values. Further, sudden increase of* Ae. aegypti* mosquito adult catches and increase of OI were observed along with the increasing number of dengue cases recorded within the study area. These findings highlighted the need for more advanced method of* Aedes* mosquito control measures in addition to the regular removal of possible* Aedes* mosquito breeding sites within a given area. Systematic review of publications within the last 10 years revealed only a 22% positive correlation between vector indices and dengue cases [18–20], whereas others reported little evidence of a quantifiable association between these two data sets [18–20]. Appropriately designed studies with larger data sets were recommended by these researchers to investigate the association between vector indices and dengue transmission that could be reliably used to predict outbreaks. Therefore, absence of significant associations between the larval indices and dengue cases or meteorological parameters and dengue cases, could be due to the limitations in the study period.

The findings of this research suggest that human movement could be a possible reason for a nonsignificant association between dengue case incidence and vector data as identified from the preliminary epidemiological and sociological survey [21]. According to above survey, most of the households were having occupants living under the lease or rent contract, which was less than 2 years. Further as previously reported by the authors [21], only a proportion of dengue infections that occurred in the study area were acquired in the individual's home environment, whereas majority of infectious were acquired outside the home environment (working place, study place, relatives place, etc.). However, the relative abundance of* Aedes* mosquito population and high human density were factors that influenced the spread of dengue within the study area once a case of dengue occurred.

According to adult, larval, and ovitrap survey data,* Ae. albopictus *was the dominant species at both indoor and outdoor environments in the study area. In other words many researchers have stated that the urbanization affected the breeding of* Ae. aegypti *and* Ae. albopictus *species differently, whereas others explained that breeding of* Ae. aegypti* is more prominent in man-made artificial containers (Tyres, Curd pots, discarded plastic cups, barrels, blocked drains, roof gutters, etc.) and* Ae. albopictus* is more prominent in vegetation or natural breeding places (e.g., tree holes, leaf axils, cut bamboo trunks, etc.) [22]. Therefore, abundance of* Ae. aegypti *is high in urban areas and* Ae. albopictus *in rural areas, whereas two species cooccur in the suburban areas [23]. A study conducted by Hawley has shown that* Ae. albopictus *is usually found outside the houses and it prefers vegetated areas and breeds in both artificial and natural water containers. Some of the findings suggested that* Ae. albopictus* could even breed in slightly polluted water as well [24]. Findings of the current study have also proved the above phenomena by always having higher OI values for outdoor placement than inside placements [16]. In another study conducted by Norzahira et al. [25] in Malaysia, similar type of findings has been reported from a suburban area, similar to the study settings of Narangodapaluwa PHI area. This was further confirmed by the findings obtained from the socioeconomic and demographic survey conducted in the same study area [16].

As stated above, the presence of* Ae. albopictus* population was high in the study area than the* Ae. aegypti*, the primary vector responsible for transmission of dengue. Further, abundance of* Ae. aegypti* was not stable throughout the study period (Figure 2). However, many researchers emphasized that an Ovitrap index above 10% for* Aedes *species in an area is an indication for a possible risk of dengue outbreak occurrence once the dengue case occurred [23, 26–28]. Mixed infestation of both* Ae. aegypti* and* Ae. albopictus* in single breeding places has also been recorded in several studies [23–28]. Similar findings were also observed in ovitraps placed both indoors and outdoors within the study site. However, the percentage of mixed infestation remained very low (0.045%), when compared to other studies [23–28].

In this study, pupal indices were calculated as recommended by the World Health Organization [22]. However, only a few pupae were recorded within both study and control sites. In case of the association between the OI and dengue cases and the meteorological data and dengue cases, the relationships remained nonsignificant. This may be due a variety of factors such as limited time frame of data consideration, migration of mosquitoes from adjacent areas to the study site, a poor reflection of actual rainfall and temperature data (since the meteorological station was out of the study site), and human migration from and to the study site. Therefore, to have more accurate reflection, a larger well designed studies are required.

Suggestions to define threshold values for the OI, which could forecast and prevent outbreaks by timely implementation of control measures, would require continuous long-term data collection for a minimum of five years [23]. Such threshold value should be tested and researched further by other workers for it to be an acceptable threshold value for OI or other entomological indices, which could be adopted by the country for dengue control operations. Based on the findings of the current study, it can be recommended that OI is highly useful to monitor the population of* Aedes* mosquitoes by detecting the presence of eggs/larvae/pupae, rather than being used as a tool for prediction of dengue outbreaks, under short-term conditions.

## 5. Conclusion

The adult mosquito populations indicated denoted temporal variations throughout the study period. However, the density of* Aedes *larvae reflected by the Breteau index remained low, <3%. The study area was positive for* Ae. albopictus* with an adult capturing range of 1~15/34 households, while the prevalence of* Ae. aegypti *remained highly restricted. The ovipositing preference of mosquitoes did not indicate any significant variations between the ovitraps placed at ground level and at a height of 1.5-2m, even though the inside and outside of the premises had a significant influence. The OI varied from 56.9% to 94.7% during the study period of 12 months, indicating two peaks at the monsoons. Further, the effect of larval indices (BI, CI, HI, and OI) and meteorological parameters on the incidence of dengue epidemics in Narangodapaluwa PHI area, was not significant (p<0.05). Migration of mosquitoes and patients and limitations in the study period could be considered as possible factors affecting the absence of a significant relationship.

## Figures and Tables

**Table 1:** Results of the initial 6-month ovitrap survey.

**Ovitrap placement**	**Study month**	**No. of larvae present**	**No. of eggs present**	**No. of eggs hatched**
ISG	June	56	2431	1103

ISG	July	235	2208	1498

ISG	August	263	1146	686

ISG	September	259	976	535

ISG	October	397	367	613

ISG	November	145	422	380

ISH	June	155	3127	1381

ISH	July	234	2144	1379

ISH	August	305	1744	969

ISH	September	274	1374	770

ISH	October	344	1451	935

ISH	November	397	1406	1023

OSG	June	1102	5501	2689

OSG	July	882	5074	3255

OSG	August	1367	3179	2015

OSG	September	1552	2976	1699

OSG	October	1601	1826	1195

OSG	November	555	1221	856

OSH	June	666	4129	1713

OSH	July	718	3234	2234

OSH	August	1610	2589	1645

OSH	September	1937	2719	1599

OSH	October	1854	1783	1207

OSH	November	1683	2408	1650

**Table 2:** Final 1-year ovitrap survey results.

**Study year**	**Study month**	**Field visit time**	**OI**	**Study year**	**Study month**	**Field visit time**	**OI**
2015	June	1	73.7	2015	November	25	78.2

2015	June	2	72.1	2015	November	26	82.2

2015	June	3	61.0	2015	December	27	80.2

2015	June	4	66.9	2015	December	28	75.0

2015	June	5	81.2	2015	December	29	72.7

2015	July	6	68.2	2015	December	30	69.8

2015	July	7	80.0	2016	January	31	68.8

2015	July	8	74.8	2016	January	32	78.1

2015	July	9	65.3	2016	January	33	71.1

2015	August	10	67.5	2016	January	34	67.6

2015	August	11	60.8	2016	January	35	76.2

2015	August	12	75.6	2016	February	36	56.9

2015	August	13	71.4	2016	February	37	57.4

2015	September	14	66.3	2016	February	38	72.9

2015	September	15	75.0	2016	February	39	70.8

2015	September	16	78.8	2016	March	40	78.0

2015	September	17	79.3	2016	March	41	67.3

2015	September	18	70.8	2016	March	42	70.8

2015	October	19	85.1	2016	March	43	94.7

2015	October	20	77.2	2016	April	44	84.9

2015	October	21	77.9	2016	April	45	92.6

2015	October	22	79.0	2016	April	46	89.7

2015	November	23	80.3	2016	April	47	89.7

2015	November	24	81.8	2016	April	48	88.4

## Data Availability

The data used to support the findings of this study are available from the corresponding author upon request.
